# Control of sexually transmitted infections and global elimination targets, South-East Asia Region

**DOI:** 10.2471/BLT.20.254003

**Published:** 2021-04-01

**Authors:** Mukta Sharma, Bharat B Rewari, Tjandra Yoga Aditama, Prasad Turlapati, Gina Dallabetta, Richard Steen

**Affiliations:** a Department of Communicable Diseases, World Health Organization Regional Office for South-East Asia, New Delhi, India.; b Hyderabad, India.; c The Bill and Melinda Gates Foundation, Washington, DC, United States of America.; d Department of Public Health, Erasmus MC, University Medical Centre Rotterdam, Dr Molewaterplein 40, 3015 GD, Rotterdam, Netherlands.

## Abstract

The World Health Organization (WHO) set targets for a 90% reduction in the incidence of syphilis and gonorrhoea between 2018 and 2030. We review trends in sexually transmitted infections in the WHO South-East Asia Region to assess the feasibility of reaching these targets. Myanmar, Sri Lanka and Thailand reported 90% or greater reductions in the incidence or prevalence of syphilis and/or gonorrhoea between 1975 and 2005. Evidence suggests that smaller, more recent reductions in trends in sexually transmitted infections in India have driven regional declines. In other countries, sexually transmitted infections remain high or are increasing or data are not reliable enough to measure change. Sri Lanka and Thailand have strong control programmes for sexually transmitted infections that ensure universal access to services for these infections and targeted interventions in key populations. India and Myanmar have implemented targeted control efforts on a large scale. Other countries of the region have prioritized control of human immunodeficiency virus, and limited resources are available for other sexually transmitted infections. At national and subnational levels, data show rapid declines in sexually transmitted infections when targeted promotion of condom use and sexually transmitted infection services are scaled up to reach large numbers of sex workers. In contrast, recent outbreaks of sexually transmitted infections in underserved populations of men who have sex with men have been linked to rising trends in sexually transmitted infections in the region. A renewed and focused response to sexually transmitted infections in the region is needed to meet global elimination targets.

## Résumé

L'Organisation mondiale de la Santé (OMS) a fixé des objectifs pour réduire à 90% l'incidence de la syphilis et de la gonorrhée entre 2018 et 2030. Nous avons étudié les tendances en matière d'infections sexuellement transmissibles dans la Région d'Asie du Sud-Est de l'OMS afin d'évaluer la faisabilité de ces objectifs. Le Myanmar, le Sri Lanka et la Thaïlande ont signalé une diminution de 90% ou plus dans l'incidence ou la prévalence de la syphilis et/ou de la gonorrhée entre 1975 et 2005. Les données semblent indiquer une tendance à la baisse plus récente et moins significative des infections sexuellement transmissibles en Inde, entraînant une décrue régionale. Dans d'autres pays, soit le nombre d'infections sexuellement transmissibles demeure élevé ou continue sa progression, soit les informations disponibles ne sont pas suffisamment fiables pour en mesurer l'évolution. Le Sri Lanka et la Thaïlande ont établi de solides programmes de lutte contre les infections sexuellement transmissibles, permettant d'accéder à des services spécialement conçus pour leur prise en charge et prévoyant une intervention ciblée au sein des populations clés. De leur côté, l'Inde et le Myanmar ont déployé des efforts à grande échelle afin de mener des actions ciblées. D'autres pays de la région ont privilégié la lutte contre le virus de l'immunodéficience humaine; pour les autres infections sexuellement transmissibles, leurs ressources sont limitées. Aux niveaux national et infranational, les données révèlent un rapide déclin des infections sexuellement transmissibles lorsque la promotion ciblée pour encourager l'usage du préservatif et les services dédiés à la prise en charge de telles affections sont renforcés afin de toucher un plus grand nombre de travailleurs du sexe. En revanche, les épidémies d'infections sexuellement transmissibles observées dernièrement au sein de populations défavorisées d'hommes ayant des relations sexuelles avec d'autres hommes ont entraîné une hausse dans la région. Il est donc indispensable d'apporter une réponse remaniée et ciblée face aux infections sexuellement transmissibles dans la région en vue d'atteindre les objectifs mondiaux d'élimination.

## Resumen

La Organización Mundial de la Salud (OMS) fijó como objetivo una reducción del 90% en la incidencia de la sífilis y la gonorrea entre 2018 y 2030. Revisamos las tendencias de las infecciones de transmisión sexual en la Región del Sudeste Asiático de la OMS para evaluar la viabilidad de alcanzar estos objetivos. Myanmar, Sri Lanka y Tailandia informaron de reducciones del 90% o más en la incidencia o prevalencia de sífilis y/o gonorrea entre 1975 y 2005. Los datos sugieren que las reducciones más pequeñas y recientes en las tendencias de las infecciones de transmisión sexual en la India han impulsado los descensos regionales. En otros países, las infecciones de transmisión sexual siguen siendo elevadas o están aumentando, o los datos no son lo suficientemente fiables como para medir el cambio. Sri Lanka y Tailandia tienen sólidos programas de control de las infecciones de transmisión sexual que garantizan el acceso universal a los servicios para estas infecciones e intervenciones específicas en poblaciones clave. India y Myanmar han implementado esfuerzos de control específicos a gran escala. Otros países de la región han dado prioridad a la lucha contra el virus de la inmunodeficiencia humana y disponen de recursos limitados para otras infecciones de transmisión sexual. A nivel nacional y subnacional, los datos muestran un rápido descenso de las infecciones de transmisión sexual cuando se amplía la promoción del uso del preservativo y los servicios para las infecciones de transmisión sexual para llegar a un gran número de profesionales del ámbito sexual. Por el contrario, los recientes brotes de infecciones de transmisión sexual en poblaciones desatendidas de hombres que tienen relaciones sexuales con otros hombres se han relacionado con las tendencias al alza de las infecciones de transmisión sexual en la región. Se necesita una respuesta renovada y centrada en las infecciones de transmisión sexual en la región para alcanzar los objetivos mundiales de eliminación.

## ملخص

وضعت منظمة الصحة العالمية (WHO) أهدافًا لخفض بنسبة 90% في الإصابة بالزهري والسيلان بين عامي 2018 و2030. نحن نقوم بمراجعة الاتجاهات في الأمراض المنقولة جنسيًا في منطقة جنوب شرق آسيا التابعة لمنظمة الصحة العالمية، لتقييم جدوى الوصول إلى هذه الأهداف. أعلنت كل من ميانمار وسري لانكا وتايلند عن انخفاضات بنسبة 90% أو أكبر في الإصابة بالزهري و/أو السيلان، وانتشارهما بين عامي 1975 و2005. وتشير الدلائل إلى أن الانخفاضات الأصغر والأحدث في اتجاهات الإصابة بالأمراض المنقولة جنسيًا في الهند قد حفزت حدوث انخفاضات إقليمية. في بلدان أخرى، تظل الأمراض المنقولة جنسياً مرتفعة، أو متجهة للازدياد، أو أن البيانات غير موثوقة بشكل كاف لقياس التغيير. يوجد لدى كل من سري لانكا وتايلند برامج قوية لمكافحة الأمراض المنقولة جنسياً والتي تضمن الوصول الشامل إلى خدمات هذه الأمراض والتدخلات المستهدفة في الفئات السكانية الرئيسية. قامت كل من الهند وميانمار بتنفيذ جهود المكافحة المستهدفة على نطاق واسع. منحت بلدان أخرى في المنطقة الأولوية لمكافحة فيروس نقص المناعة البشرية، مع توفير موارد محدودة للأمراض الأخرى المنقولة جنسياً. على المستويين الوطني ودون الوطني، توضح البيانات انخفاضًا سريعًا في الأمراض المنقولة جنسياً عندما يتم توسيع نطاق الترويج المستهدف لاستخدام الواقي الذكري، وخدمات الأمراض المنقولة جنسياً للوصول إلى أعداد كبيرة من المشتغلين بالجنس. في المقابل، فإن الحالات الحديثة لتفشي الأمراض المنقولة جنسياً بين فئات السكان المحرومين من الخدمات من الرجال الذين يمارسون الجنس مع الرجال، قد ارتبطت بالاتجاهات المتزايدة في الأمراض المنقولة جنسياً في المنطقة. هناك حاجة إلى استجابة متجددة ومركزة للأمراض المنقولة جنسياً في المنطقة، وذلك بهدف تلبية الأهداف العالمية للقضاء على الأمراض.

## 摘要

世界卫生组织 (WHO) 制定了目标，计划在 2018 年至 2030 年期间将梅毒和淋病发病率降低 90%。我们回顾了 WHO 东南亚地区性传播感染的趋势，以评估实现这些目标的可行性。缅甸、斯里兰卡和泰国报告在 1975 年至 2005 年期间梅毒和/或淋病的发病率或患病率下降了 90% 以上。有证据表明，印度性传播感染趋势近期出现较小幅度的降低，这一结果推动了地区性疾病的减少。在其他国家，性传播感染仍居高不下，或者正在增加，或者数据不足以衡量变化。斯里兰卡和泰国针对性传播感染制定了强有力的控制计划，确保普及针对这些感染的服务，并在关键人群中采取针对性干预措施。印度和缅甸大规模实施了有针对性的防控工作。该地区的其他国家已将人类免疫缺陷病毒的控制列为优先工作事项，用于其他性传播感染的资源有限。数据显示，在国家和国家以下各级层面，当有针对性地推广使用避孕套和扩大针对性传播感染的服务，以便可以惠及大量性工作者时，性传播感染率迅速下降。相比之下，最近在医疗资源不足地区的男男性行为者中爆发的性传播感染，与该地区性传播感染的上升趋势有一定的联系。为了实现全球消除目标，需要针对该地区的性传播感染侧重点重新制定新的应对措施。

## Резюме

Всемирная организация здравоохранения (ВОЗ) определила цели по снижению заболеваемости сифилисом и гонореей на 90% в период с 2018 по 2030 год. Авторы анализируют тенденции распространения инфекций, передаваемых половым путем, в регионе Юго-Восточной Азии ВОЗ, чтобы оценить возможность достижения этих целей. Мьянма, Таиланд и Шри-Ланка сообщили о сокращении заболеваемости или распространенности сифилиса и (или) гонореи в период с 1975 по 2005 год на уровне 90% или выше. Факты свидетельствуют о том, что недавнее не такое масштабное снижение тенденций инфекций, передаваемых половым путем в Индии, привело к снижению на региональном уровне. В других странах число инфекций, передаваемых половым путем, остается высоким или продолжает расти либо данные недостаточно надежны для измерения изменений. В Таиланде и Шри-Ланке действуют сильные программы по борьбе с инфекциями, передаваемыми половым путем, которые обеспечивают всеобщий доступ к услугам по лечению этих инфекций и адресным мероприятиям для ключевых групп населения. В Индии и Мьянме были предприняты широкомасштабные адресные усилия по борьбе с этими инфекциями. Другие страны региона уделяют приоритетное внимание борьбе с вирусом иммунодефицита человека, а на борьбу с другими инфекциями, передаваемыми половым путем, выделяются ограниченные ресурсы. На национальном и субнациональном уровнях данные свидетельствуют о быстром сокращении инфекций, передаваемых половым путем, когда адресное продвижение использования презервативов и услуг по борьбе с инфекциями, передаваемыми половым путем, расширяется с целью охвата большего числа работников секс-индустрии. И наоборот, недавние вспышки инфекций, передаваемых половым путем, среди не охваченных услугами групп мужчин, имеющих половые контакты с мужчинами, были связаны с тенденциями регионального роста инфекций, передаваемых половым путем. Для достижения глобальных целей по ликвидации инфекций, передаваемых половым путем, в регионе необходимы обновленные и целенаправленные ответные меры.

## Introduction

The World Health Organization’s (WHO’s) global health-sector strategy on sexually transmitted infections (2016–2021) has set targets for 90% reductions in the incidence of *Treponema pallidum* (syphilis) and *Neisseria gonorrhoeae* (gonorrhoea) infections between 2018 and 2030.^1^ Here, we review trends in sexually transmitted infections and the experiences of countries in the WHO South-East Asia Region to determine the current status of control programmes for sexually transmitted infections and the feasibility of reaching the global targets. We focus primarily on control of common curable sexually transmitted infections such as syphilis, gonorrhoea and to a lesser extent *Chlamydia trachomatis* (chlamydia) and *Haemophilus ducreyi* (chancroid) infections.

Historically, sexually transmitted infections have been among the most serious public health problems in the WHO South-East Asia Region, with substantial associated morbidity, mortality, disability and adverse pregnancy outcomes.^2^^–^^4^ The incidence and prevalence of curable sexually transmitted infections were high in urban areas and along migrant and trucking routes, and were closely linked to the rapid early spread of human immunodeficiency virus (HIV), particularly ulcerative chancroid and syphilis. However, large-scale preventive measures that focused on sex work in several countries of the region during the 1990s and early 2000s led to substantial declines in sexually transmitted infections and the slowing of the HIV epidemic.^2^^–^^4^

Recent reports of increases in the incidence of syphilis and gonorrhoea raise concerns about the adequacy of the current efforts to prevent sexually transmitted infections as control programmes focus increasingly on HIV-specific interventions such as antiretroviral therapy and pre-exposure prophylaxis without maintaining investment in primary prevention.^5^^–^^7^ Declining use of condoms and behavioural risk compensation (that is, riskier behaviour that may dilute or offset preventive benefits), particularly in key populations, may facilitate a resurgence in transmission of sexually transmitted infections at a time when primary prevention, condom programming and basic services for sexually transmitted infections are underresourced.

In this paper, we assess the current epidemiology and control of sexually transmitted infections in the WHO South-East Asia Region. We base our assessment on a 2018 report on the elimination of sexually transmitted infections in the region and other recent data.^8^ In countries with improved infection control, we discuss what constitutes an effective response to sexually transmitted infections. We consider the challenges faced by programmes for sexually transmitted infections and what actions may help to counter those challenges.

## Historical trends

WHO global estimates for four common curable infections – syphilis, gonorrhoea, chlamydia and trichomoniasis – have shown little change over three decades.^9^^–^^13^ However, the proportion of new cases of sexually transmitted infections estimated for the WHO South-East Asia Region has declined by two thirds, from 118 million (35% of the global estimate) in the 1990s to 39 million (11% of the global estimate) in 2012.^8^ Despite limitations in the methods used for these estimations, such a large magnitude of change requires further analysis to assess whether sexually transmitted infections have indeed declined in the South-East Asia Region relative to other WHO regions. 

**Fig. 1:**
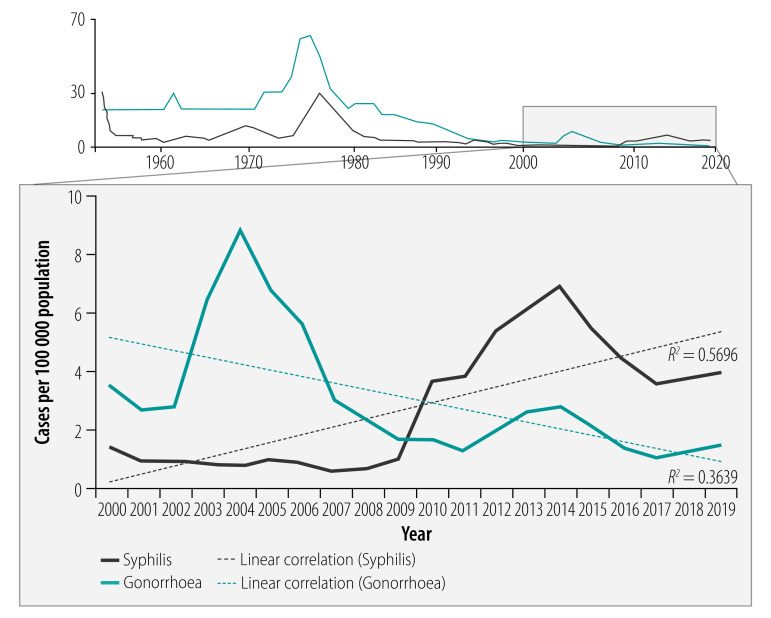
Trends in sexually transmitted infections, Sri Lanka, 1952–2019

Source: National STD/AIDS Control Programme, Colombo, Sri Lanka.

**Fig. 2:**
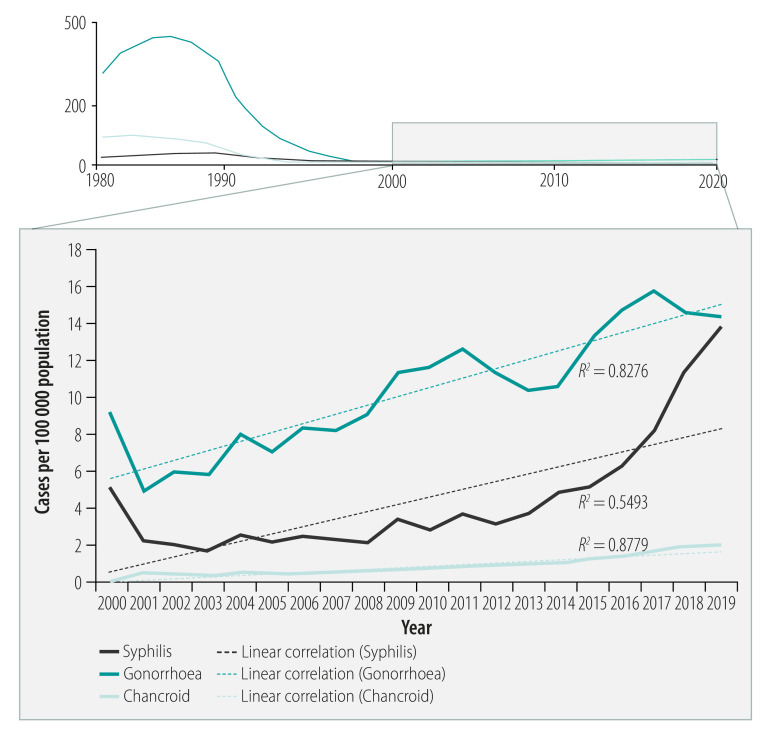
Trends in sexually transmitted infections, Thailand, 1982–2019

Source: Bureau of Epidemiology, Department of Disease Control, Ministry of Public Health, Bangkok, Thailand.

Although routinely reported surveillance data on sexually transmitted infections vary in completeness and reliability across the region, national data from Myanmar, Sri Lanka and Thailand, obtained from their health ministries, are good enough to examine long-term trends. In these countries, sexually transmitted infections have decreased by more than 90%.^8^ For example, in Sri Lanka, the incidence of gonorrhoea decreased from 61.6 to 3.5 cases per 100 000 population (94.3% reduction) between 1975 and 2000 (Fig. 1), while in Thailand, the incidence decreased from 445 to 7 cases per 100 000 population (98.4% reduction) between 1985 and 2005 (Fig. 2). In Myanmar, the incidence of gonorrhoea decreased from 15.4 to 1.4 per 10 000 male population (90.6% reduction, based on non-rounded figures) between 1985 and 2005. In addition, in Sri Lanka between 1975 and 2000, the incidence of syphilis decreased from 21.5 to 1.4 cases per 100 000 population (93.5% reduction) (Fig. 1), and in Thailand between 1985 and 2005 the incidence decreased from 32.0 to 2.1 cases per 100 000 population (93.4% reduction) (Fig. 2). In Myanmar between 1996 and 2016, the prevalence of syphilis among women attending antenatal clinics decreased from 4.2% to 0.3%, a 92.9% reduction. Large reductions in the incidence of chancroid were also reported between 1985 and 2005, from 93.0 to 0.4 cases per 100 000 population (99.6% reduction) in Thailand (Fig. 2). and from 7.5 to 0.4 cases per 10 000 male population (94.5% reduction, based on non-rounded figures) in Myanmar.

Evidence from many sources suggests that smaller reductions in sexually transmitted infections in India’s large population – more than two thirds of the region’s total population – have driven regional declines.^3^^,^^4^ However, reporting on sexually transmitted infections in India is not consistent enough to detect trends as is the case in Sri Lanka and Thailand. On the other hand, many epidemiological studies, particularly over a period of increased investment in control of sexually transmitted infections (including the large Avahan India AIDS Initiative), show changing trends and patterns in the incidence and prevalence of sexually transmitted infections in India.^14^^,^^15^ Studies from the 1990s in India indicate poor control, with a high prevalence of bacterial and ulcerative sexually transmitted infections including chancroid, concentration of these infections in urban areas linked to migration and mobility, and strong associations with HIV acquisition and transmission.^16^^–^^20^ Later studies show a very different picture, with evidence of large declines in sexually transmitted infections between the late 1990s and 2010 among key populations (sex workers, men who have sex with men, people who use drugs and prisoners); male bridge groups (higher risk men, e.g. migrants or transport workers, who have contact with both key populations and lower-risk populations); and pregnant women.^21^^–^^42^ Taken together, these studies provide additional evidence of declining trends, closely linked to intervention efforts, and of an epidemiological transition from predominately bacterial to viral sexually transmitted infections.^21^^–^^42^ The strongest intervention-linked data show declines in the incidence or prevalence of sexually transmitted infections between 21.3% (from 8.9% to 7.0%) and 77.3% (from 9.7% to 2.2%) across several large Indian states between 2004 and 2010.^36^^,^^41^

Some of the earliest and largest decreases in sexually transmitted infections were reported among female sex workers in Kolkata, India. The prevalence of syphilis, assessed by sentinel surveillance, declined by more than 99%, from more than 25% in 1992 to 0.2% in 2005.^43^ Another community-led intervention among sex workers in Karnataka, India, reported significant reductions in the prevalence of syphilis (45.4%, from 24.9% to 13.6%), gonorrhoea (83.3%, from 5.4% to 0.9%) and chlamydia (63.0%, from 10.8% to 4.0%) from the start of the intervention in 2004 until 2009 – with more recent routine clinical data suggesting near elimination of symptomatic cases of sexually transmitted infections.^22^^,^^23^^,^^25^ Increasing condom use and statistically significant declines in sexually transmitted infections and HIV were reported in surveys from other districts in Karnataka from 2004 to 2011.^24^^,^^25^ Large reductions in syphilis and HIV prevalence were also reported in pregnant women over the same period.^23^^,^^44^

Among the remaining seven countries in the South-East Asia Region, routine surveillance of sexually transmitted infections is neither complete nor reliable enough to assess trends and few studies are reported in the literature. Where declines in sexually transmitted infections have been documented in a few specific locations, these reductions are linked to programmes that have increased condom use in sex work while also improving clinical services for sexually transmitted infections, or are associated with specific interventions for sexually transmitted infections such as periodic presumptive treatment. In Bangladesh, for example, a community-based randomized controlled trial reported an 83% decrease (from 41% to 7%) in the prevalence of either gonorrhoea or chlamydia in female sex workers 9 months after a periodic presumptive treatment intervention and enhanced syndromic management.^45^ In Bintan Island, Indonesia, the prevalence of gonorrhoea and/or chlamydia decreased by 78.9% (from 36.1% to 7.6%, *P* < 0.01) in female sex workers over 15 months in 2008–2009, with a lower prevalence among those who received periodic presumptive treatment (*P* < 0.01).^46^ Of note, the prevalence of consistent condom use reported in this study doubled to 40% *(P <* 0.01). Elsewhere in Indonesia, the prevalence of active syphilis (defined as rapid plasma reagin ≥ 1:8) was 35.0% lower (3.9% after the intervention compared with 6.0% before) in 10 cities between 2005 and 2007 among those who received at least one dose of periodic presumptive treatment (*P* = 0.008).^47^

## Current trends

The large decreases in sexually transmitted infections documented in several countries of the region from 1980 to 2010 can be at least partly attributed to comprehensive prevention efforts – in particular, promotion of condom use in targeted high-risk groups and programmes to control sexually transmitted infections – in response to the rapidly growing HIV epidemics. During the past decade, however, these trends appear to have levelled off or reversed in at least some populations, with a growing number of outbreaks of sexually transmitted infections being reported or rising trends being seen across the region.^5^^–^^7^^,^^48^ The importance of reliable surveillance of sexually transmitted infections and strong programme capacity is evident in the context of this recent resurgence. Countries with reliable national surveillance have been able to detect rising trends of sexually transmitted infections at the national level, while evidence from other countries is limited to surveys conducted in a few locations in specific population groups. In Thailand and Sri Lanka, reported syphilis cases rose on average 2.4 and 5.1 times, respectively, from 2000–2009 to 2010–2019, while gonorrhoea cases increased 1.7 times in Thailand over the same periods (Fig. 1 and Fig. 2).

While rebounding transmission of sexually transmitted infections is a challenge, overall rates in Sri Lanka and Thailand are still low compared with historical trends, and both countries have recently been validated as having achieved elimination of mother-to-child transmission of HIV and congenital syphilis.^1^ Viewed in a broader context comparing rates across regions, the incidence rates of syphilis and gonorrhoea in Thailand (respectively, 8.2 and 15.8 cases per 100 000 population) and Sri Lanka (respectively, 3.6 and 1.1 cases per 100 000 population) in 2017 were comparable to those reported in Europe (7.1 and 22.2 cases per 100 000 population).^49^^,^^50^

Elsewhere in the region, the trends in sexually transmitted infections are less clear. In Indonesia, trend data from integrated biological and behavioural surveys in key populations show high and stable prevalence rates of gonorrhoea and chlamydia between 2009 and 2019, with no sign of a downward trend. The prevalence of syphilis in Jakarta and Bandung increased 3–5 times between 2007 and 2018 in men who have sex with men and transgender groups. Syphilis rates were lower but also increasing in people who inject drugs over the same period.^51^

## Components of a strong response

Reflecting on the elements of good control of sexually transmitted infections in the South-East Asia Region, it is striking that countries that have invested in four key areas have shown much better progress compared with countries that have not.^51^

First, the responses of the countries that have made progress have been data driven, and investment in sexually transmitted infection surveillance systems and programme monitoring has been maintained and linked to antibiotic resistance monitoring.

Second, countries that have ensured universal access to screening, diagnosis and treatment of syphilis and HIV for pregnant women attending antenatal care (such as Bhutan, Maldives, Sri Lanka and Thailand) have been able to reduce the prevalence of these diseases in pregnancy more than countries that have not provided such access. Furthermore, successful access has been obtained through collaboration across primary care, reproductive health and maternal and child health services.

Third, these countries have had a strong focus on key population interventions and, apart from screening, diagnosis and treatment of HIV, they have made large investments in primary prevention. As part of prevention, not only have these countries improved the provision of condoms and lubricants, they have also ensured access to regular sexually transmitted infection screening and tackled structural drivers of risk and vulnerability. For example, Thailand’s 100% condom-use programme put the responsibility for condom use on the client and ensured that brothel owners had a duty to ensure safe sex practices.^2^ Furthermore, community voices and concerns were heard and integrated into programme design and service delivery models in Bangladesh, India and elsewhere.^21^^,^^22^

Fourth, and perhaps most importantly, countries that have ensured adequate resources for a decentralized programme within the context of access to universal health coverage have been more effective in controlling sexually transmitted infections. Having a separate and clearly articulated strategy to control sexually transmitted infections and substantial domestic investments have helped secure adequate resources. For example, the programme for the elimination of mother-to-child transmission of HIV and syphilis in Sri Lanka builds on the strong foundations of public health and primary health care services that have operated for several decades: Sri Lanka started its so-called antivenereal disease campaign in 1952, and the National sexually transmitted disease and AIDS control programme was established in 1987, with a key objective of preventing HIV and sexually transmitted infections in the community. The programme for the elimination of mother-to-child transmission of HIV and syphilis is funded entirely by the Ministry of Health of Sri Lanka. Similarly, in Thailand, the National Health Security Office ensures access to syphilis screening and treatment not just for all pregnant mothers, but also for key populations. In India, a large targeted intervention programme working with key populations in the community is funded through domestic investments and has been delivered on a large scale for over 20 years.^8^

Looking ahead, these elements of effective control of sexually transmitted infections are still relevant. However, risk behaviours have changed, particularly through the use of social media and the Internet by many young and key populations. These changes challenge the effectiveness of facility-based programming in preventing and treating sexually transmitted infections. To respond to this challenge requires programmes to innovate and explore the potential of digital health tools.

Many services for sexually transmitted infections have depended heavily on HIV funding, which is likely to become less plentiful in the future. With less funding, sexually transmitted infection services will need to find opportunities for integration and efficiency. Integrating regular sexually transmitted infection screening in men’s health clinics and moving towards newer testing approaches, such as self-testing and provider-initiated testing, is one approach. Other options include making use of the triple elimination agenda for the elimination of mother-to-child transmission of HIV, syphilis and hepatitis B infection, as well as dual diagnostic kits for HIV and syphilis testing, point-of-care tests and molecular diagnostic testing.

Control of sexually transmitted infections cannot be achieved only through a government-led response in the South-East Asia Region, because the private sector is responsible for a large proportion of health-care provision, and out-of-pocket health expenditure is high. Therefore, including screening and treatment of sexually transmitted infections as part of a minimum essential service package for outpatients, provided by social health insurance through an accredited service provider (public, private or nongovernmental), would go a long way to improving access and provider choice. As countries such as India and Indonesia move forward in improving social health insurance, programmes to control sexually transmitted infections have the opportunity to make use of the wider health system and domestic funding.

## Conclusion

Outstanding, although uneven, progress has been made in controlling sexually transmitted infections in the South-East Asia Region over the years. In several countries, this progress has been sustained so that elimination of several sexually transmitted infections has become a possible objective. However, while historical trends support the feasibility of reducing the incidence of syphilis and gonorrhoea by 90%, new challenges have emerged to reaching these global elimination targets.

For countries such as Sri Lanka and Thailand which have well established programmes for control of sexually transmitted infections, new strategies will clearly be needed to address rebounding transmission. Since the global targets for syphilis and gonorrhoea use 2018 as the baseline, reducing incidence further will require more intensive case finding to identify small clusters and interrupt transmission early. Disease elimination is always most difficult at this final stage when cases become rare and more difficult to detect. It is worth noting that further reductions in the incidence of syphilis and gonorrhoea in Sri Lanka and Thailand would bring their rates to lower than the rates of many high-income countries. 

Countries with weaker control programmes for sexually transmitted infections should be motivated by knowing that the global 90% reduction targets for syphilis and gonorrhoea are feasible, particularly for syphilis. Data on syphilis are more available and reliable than for other sexually transmitted infections because of inexpensive diagnostics and, in countries with limited capacity to diagnose other sexually transmitted infections, tracking syphilis trends is useful for monitoring overall control efforts for sexually transmitted infections. Evidence of a decrease in syphilis, as well as progress in eliminating mother-to-child transmission of the infection in several countries, supports the feasibility of regional elimination of syphilis as a public health problem. Weak surveillance of sexually transmitted infections and limited syphilis screening of key populations and pregnant women are the main barriers to elimination of syphilis in the region. Recent increases in syphilis among men who have sex with men in several countries of the region and elsewhere underline the importance of routinely screening key populations for this disease and other sexually transmitted infections, and monitoring prevalence trends. Such outbreaks and rebounding trends, together with increasing antimicrobial resistance, are the main new challenges for countries aiming to reach the global targets for elimination of sexually transmitted infections.

A focused and prioritized response targeting the most important sexually transmitted infections from a public health perspective is clearly needed now. To move this agenda forward, sexually transmitted infections need to become visible again. Political attention to these infections has been half-hearted at best and this is compounded by the lack of any specific targets within the sustainable development goals that address sexually transmitted infections. Raising awareness of the large and preventable disease burden of sexually transmitted infections and its effect on people and societies will require not just data, but also investment to generate demand for services and high-level advocacy.
